# Great Western Sanitary Fair

**Published:** 1863-12

**Authors:** 


### GREAT WESTERN SANITARY FAIR.

Though this subject does not specially pertain to dentistry, yet we can not refrain from making a brief record of it; for it is one of the great events of this city, and, indeed, of this part of the country. It was a most magnificent affair, both in the conception and execution. The organization was hastily effected, and notwithstanding this, was very perfect and efficient. The execution of the plan, in all the details and minutiæ, was very thorough and rapid, perhaps more so than was ever before realized in a similar enterprise. There seemed to be no jarring in the operation of machinery,—all was perfect harmony. It was controlled by master-minds, and carried on by the indefatigable enterprise and labor of all concerned. It was a great work, based upon love and benevolence.

As an outpouring of the great popular heart, for patriotism and liberty, and in behalf of suffering humanity, it has no parallel. Its moral effect upon all who are engaged, and even upon those who are mere lookers-on, will be very great; but the greatest good that will result, will be the cheer and gladness that it will carry to the hearts of the weary war-worn soldiers. And may every dollar of this immense contribution go to our noble defenders as a messenger of love, baptized with the warmest sympathies of loving friends at home. T.

